# Differences in SpeB protease activity among group A streptococci associated with superficial, invasive, and autoimmune disease

**DOI:** 10.1371/journal.pone.0177784

**Published:** 2017-05-17

**Authors:** Anhphan T. Ly, John P. Noto, Odaelys L. Walwyn, Robert R. Tanz, Stanford T. Shulman, William Kabat, Debra E. Bessen

**Affiliations:** 1 Department of Microbiology and Immunology, New York Medical College, Valhalla, New York, United States of America; 2 Department of Pediatrics, Northwestern University Feinberg School of Medicine and Ann & Robert H. Lurie Children's Hospital, Chicago, Illinois, United States of America; Ross University School of Medicine, DOMINICA

## Abstract

The secreted cysteine proteinase SpeB is an important virulence factor of group A streptococci (GAS), whereby SpeB activity varies widely among strains. To establish the degree to which SpeB activity correlates with disease, GAS organisms were recovered from patients with pharyngitis, impetigo, invasive disease or acute rheumatic fever (ARF), and selected for analysis using rigorous sampling criteria; >300 GAS isolates were tested for SpeB activity by casein digestion assays, and each GAS isolate was scored as a SpeB-producer or non-producer. Highly significant statistical differences (*p* < 0.01) in SpeB production are observed between GAS recovered from patients with ARF (41.5% SpeB-non-producers) compared to pharyngitis (20.5%), invasive disease (16.7%), and impetigo (5.5%). SpeB activity differences between pharyngitis and impetigo isolates are also significant, whereas pharyngitis versus invasive isolates show no significant difference. The disproportionately greater number of SpeB-non-producers among ARF-associated isolates may indicate an altered transcriptional program for many rheumatogenic strains and/or a protective role for SpeB in GAS-triggered autoimmunity.

## Introduction

Group A *Streptococcus* (GAS) is a human pathogen of global importance that most often causes a superficial self-limiting infection at the throat (pharyngitis) or skin (impetigo), leading to ~750 million infections per year [1]. GAS is also associated with high rates of morbidity and mortality due to invasive and autoimmune disease, although these conditions are far less prevalent. Acute rheumatic fever (ARF) follows an inadequately treated GAS throat infection by a so-called "rheumatogenic" strain, and can often lead to rheumatic heart disease via autoimmune attack of heart valves [2, 3]. The existence of distinct "rheumatogenic" and "non-rheumatogenic" strains of GAS has been long recognized [4–7], but their distinguishing properties are not well-understood. Numerous other GAS strains are often considered to be “disease specialists” [8–12].

The secreted cysteine protease SpeB is a key virulence factor of GAS that acts by degrading human proteins having a protective role in host defense; SpeB also targets many extracellular proteins produced by GAS (reviewed in [13, 14]). Although virtually all GAS isolates harbor a *speB* gene, SpeB activity can vary widely among strains; *speB* expression is modulated by several global regulators of transcription and importantly, it is tightly coordinated with expression of a large number of other genes (reviewed in [15]).

Studies by one group of investigators show that SpeB activity correlates with GAS disease, whereby a substantial fraction (~41%) of severe invasive disease isolates harboring *emm1* (M-type 1 or M1) fail to produce SpeB in vitro [16]. A direct role in the transition from localized to invasive disease has been elucidated for (a lack of) SpeB and/or co-transcribed genes [13, 17–19]. In a mouse model for invasive disease, mutants with defects in a two-component regulatory system (CovRS) and having decreased SpeB production are more virulent [20]. Possible conflicting findings on the association of SpeB activity with GAS recovered from patients with invasive disease—both the mild and severe forms combined—have also been reported. In this more recent study, the vast majority (>80%) of both invasive and pharyngitis *emm1* isolates are SpeB-producers, as are invasive isolates of three other *emm* types (*emm28*, *emm59*, *emm89*), and there is no significant difference between the invasive versus pharyngitis isolates in terms of SpeB activity [21].

In this report, the relative distribution of a SpeB-producer phenotype is measured for 322 GAS isolates recovered from patients with pharyngitis, impetigo, invasive disease or ARF. Strain sampling follows a strict set of criteria that aims to be representative of the organisms found within patient populations (i.e., population-based). Importantly, the sampling criteria also captures a very wide range of genetic diversity among GAS isolates, as defined by the *emm* gene marker.

## Results

### Diversity and characteristics of the GAS strain populations

Study sample sets of GAS isolates associated with four distinct clinical conditions were assembled: ARF, pharyngitis, impetigo and invasive disease. All ARF and pharyngitis isolates were recovered from the upper respiratory tract (URT) of human subjects. Strain sampling followed strict and well-defined criteria, with the goals of assembling a genetically diverse and representative set (Table 1; Supplementary Data S1 Table). Selection of a small number of isolates sharing the same *emm* type and recovered from the same community reduces the potential skewing effects of highly prevalent clones; this was done for ARF and impetigo isolates, and one pharyngitis collection. All ARF, pharyngitis and invasive disease isolates were recovered from the United States, whereas impetigo isolates had a worldwide distribution.

**Table 1:** Characteristics and diversity of GAS isolates under analysis.

Disease group	No. of isolates	Dates	Geographic origin	No. of *emm* types represented	*D*, based on *emm* type ^a^	No. of *emm* subtypes represented	*D*, based on *emm* subtype
Pharyngitis	78	2001–2002	Bristol, CT	19	0.8998	27	0.9261
Pharyngitis	68	2012	Chicago, IL	14	0.9166	16	0.9289
***Total pharyngitis***	146	n/a	n/a	21	0.9125	34	0.9369
Impetigo	58	1994–1996	Australia	28	0.9782	29	0.9794
Impetigo	16	1971–1988	Worldwide	16	1.0000	16	1.0000
***Total impetigo***	74	n/a	n/a	39	0.9852	41	0.9863
Invasive	60	1995	CT	20	0.8667	n.d.	n.d.
ARF	42	1933–1989	USA	20	0.9489	34	0.9907
***Total isolates***	322	n/a	n/a	69	0.9530	96	0.9811

^a^ *D*, Simpson's diversity index

The genetic markers used for analysis of strain diversity are *emm* type and *emm* subtype. Data show that the sampled selections of GAS isolates display very high levels of genetic diversity, with Simpson diversity index (*D*) values approaching one, signifying that most isolates are distinct (Table 1). The relative diversity *D* values show impetigo > ARF > pharyngitis > invasive isolates (based on *emm* type), and ARF > impetigo > pharyngitis isolates (based on *emm* subtype; *emm* subtype was not determined for invasive isolates).

For the 322 GAS isolates under study, 69 distinct *emm* types are represented (Table 1), accounting for ~30% of the known *emm* types of the *Streptococcus pyogenes* species [22]. Of the 42 ARF-associated isolates, 57% harbor an *emm* type that is shared with the pharyngitis isolates (S2 Table), consistent with ARF having extensive overlap with other URT strains. Of the 60 invasive disease isolates (from CT in 1995), 88% have an *emm* type that is also shared with the pharyngitis isolates, consistent with invasive disease isolates being largely reflective of the prevailing pharyngitis *emm* types within a region [23, 24]. In sharp contrast, only 19% of the 74 impetigo isolates have an *emm* type that is shared with the pharyngitis isolates, and even fewer have an *emm* type present among the ARF-associated (9%) or CT invasive disease (9%) isolates (S2 Table).

Putative rheumatogenic M protein types in the United States include M-types 1, 3, 5, 6, 14, 18, 19, 24, 27 and 29 [6]. These 10 *emm* types account for 24 (57%) of the 42 ARF-associated and 59 (40%) of the 146 pharyngitis isolates examined (S1 Table), which is a slight but statistically non-significant enrichment of putative rheumatogenic *emm* types among the ARF-associated isolates. Collectively, the 10 putative rheumatogenic *emm* types account for 62% of the invasive disease isolates, similar to the value for ARF-associated isolates but with a strikingly different distribution for *emm* types 1 and 18, whereby *emm1* is highly enriched in the invasive disease set (representing 30% of invasive disease isolates) and *emm18* is enriched in the ARF-associated pool (representing 14% ARF isolates). Only 4% of the impetigo isolates had a putative rheumatogenic *emm*-type as defined by [6].

### Clinical correlates of SpeB activity

SpeB activity was measured by digestion of casein following growth of GAS on agar (Table 2, S1 Table). For Columbia agar containing skim milk powder (Columbia-SM), data show highly significant statistical differences (*p* < 0.01; Fisher's exact test, 2-tailed) for SpeB-non-producer isolates recovered from patients with pharyngitis (20.5% non-producers) versus ARF (41.5%) or impetigo (5.5%). However, the difference in SpeB production for invasive (16.7% non-producers) versus pharyngitis isolates is non-significant. Data also show significant statistical differences for SpeB-non-producer isolates recovered from patients with ARF versus invasive disease (*p* = 0.011) or impetigo (*p* < 0.0001).

**Table 2:** SpeB activity phenotype correlates with GAS disease.

	Columbia agar with skim milk ^a^				C-broth agar with casein (*extrapolated values*) ^c^			
Disease group	No. of SpeB- producers	No. of SpeB- non-producers	% SpeB- non-producers	*p* value, pharyngitis versus ^b^:	No. of SpeB- producers	No. of SpeB-non-producers	% SpeB- non-producers	*p* value, pharyngitis versus ^b^:
Pharyngitis	116	30	20.5	n/a	*106*	*32*	*23*.*7*	n/a
Impetigo	69	4	5.5	0.0029	*69*	*4*	*5*.*5*	*0*.*0009*
Invasive	50	10	16.7	N.S.	44	12	21.4	*N*.*S*.
ARF	24	17	41.5	0.0085	20	20	50.0	*0*.*0016*

^a^ One ARF and one impetigo isolate had intermediate ("weak") zones of clearance on Columbia-SM agar (S1 Table), and are excluded from the calculations.

^b^ Fisher's exact test, 2-tailed; N.S., non-significant.

^c^ Data extrapolations for the complete set of GAS organisms are made for impetigo and pharyngitis isolates (*italics*). Organisms with intermediate ("weak") zones of clearance on CBrothMg-C agar (S1 Table) are excluded from the calculations.

The ARF-associated isolates were collected over a period extending >5 decades. Of the 15 isolates collected before the widespread use of penicillin (pre-1950), 33% (5 of 15) were SpeB-non-producers on Columbia-SM agar, as compared to 46% (12 of 26) of isolates collected between 1950 and 1989; this slight distinction between collection periods is not statistically significant. Similarly, 33% of the ARF-associated isolates collected during the 1980s were SpeB non-producers, as compared to 45% collected prior to 1980 (*p* = N.S.). Thus, there are no apparent effects of widespread antibiotic usage, long-term laboratory storage and/or shifting epidemiologic patterns on SpeB production by the sample set of ARF-associated organisms.

Of the 74 impetigo isolates, 58 originate from an Aboriginal Australian community located within a larger geographic region that is well-known for its high prevalence of rheumatic heart disease [25, 26]. Of the Australian GAS isolates, 7% were SpeB non-producers on Columbia-SM agar, as compared to none of the 16 impetigo isolates originating from worldwide sources (S1 Table); however, the difference between the two impetigo collections was not statistically significant (*p* = 0.5699; Fisher’s exact test, 2-tailed). The extent to which an impetigo infection triggers ARF (if at all) is unresolved [3], and the idea remains somewhat speculative. Importantly, the data show that Aboriginal Australian impetigo isolates do not resemble the ARF-associated URT isolates recovered from the United States insofar as SpeB phenotype. It can be difficult to disentangle geography from GAS disease since impetigo is primarily a disease of the tropics, and URT infection is highly prevalent in temperate regions (S2 Table).

Nine of the invasive disease isolates studied were recovered from patients with "severe" disease (i.e., streptococcal toxic shock syndrome, necrotizing fasciitis and/or pyomyositis) [23]. However, only one of these isolates (11%) failed to produce SpeB on Columbia-SM agar (data not shown). Of the invasive disease isolates recovered from the blood, 19% were SpeB-non-producers (data not shown), a value that reflects the larger set of invasive disease strains. Thus, there is no clear evidence for a skewed distribution of SpeB non-producers among clinically-defined subsets of invasive disease isolates, albeit the sample sizes are rather small.

To establish that caseinolytic activity is due to SpeB, and not attributable to other proteases, E64 (a SpeB-specific inhibitor) was added to Columbia-SM agar. All 40 SpeB producer strains tested lack caseinolytic activity in the presence of 10 μM E64 (S1 Table).

C-Broth-based agar containing casein (CBrothMg-C agar) was also used to test for SpeB activity, whereby C-Broth is an optimized formulation for high levels of SpeB production that is protein-rich and carbohydrate-poor [27]. With CBrothMg-C agar, the differences in casein digestion for GAS recovered from pharyngitis versus ARF or impetigo are even more highly significant than that observed for Columbia-SM agar (extrapolated values; Table 2). Data show highly significant statistical differences for SpeB-non-producer isolates recovered from patients with ARF (50%) versus invasive disease (21.4% non-producers; *p* = 0.0045, Fisher's exact test, 2-tailed) or impetigo (5.5%; *p* < 0.0001). However, differences between pharyngitis versus invasive disease isolates remain non-significant with CBrothMg-C agar. Although the initial goal in using C-broth-based agar was to further maximize SpeB production, this proved not to be the case: 81% of GAS produced SpeB on Columbia-SM agar, as compared to slightly fewer (78%) isolates on CBrothMg-C agar (Table 2).

Strain diversity within each clinically distinct sub-group of SpeB-producers and non-producers (on Columbia-SM agar) is extensive. The 17 ARF-associated SpeB-non-producers are represented by 11 distinct *emm* types and 15 *emm* subtypes (S1 Table); similarly, the 24 ARF-associated SpeB-producers are represented by 13 distinct *emm* types and 21 *emm* subtypes. The 10 SpeB-non-producers recovered from cases of invasive disease are also highly diverse, represented by seven distinct *emm* types. The 30 SpeB-non-producers recovered from patients with pharyngitis are somewhat less diverse than their ARF and invasive counterparts, represented by 10 distinct *emm* types and 14 *emm* subtypes. Taken together, a lack of SpeB activity is observed for strains representing a wide range of genotypes for each of the clinical disease groups.

### Concordance between different casein digestion assays

The concordance of SpeB phenotypes was examined for GAS strains tested on multiple culture medium types. For 35 GAS isolates that were SpeB-non-producers on Columbia-SM agar, nearly all (33, or 94%) were also SpeB-non-producers when tested on CBrothMg-C agar; one non-producer strain displayed intermediate zones of clearance on CBrothMg-C agar. For 166 isolates that were SpeB-producers on Columbia-SM agar, when tested on CBrothMg-C agar, 91% were also SpeB-producers, 5% had intermediate zones of clearance, and 4% were non-producers. Overall, 201 isolates were tested on both Columbia-SM and CBrothMg-C agar, and 91% were concordant in their SpeB findings. Excluding strains with intermediate zones of clearance, overall concordance between Columbia-SM and CBrothMg-C agar for SpeB production was 98.5%.

SpeB activity was also compared for 152 strains grown both on Columbia-SM agar and in C-Broth liquid medium, via the azocasein assay [27]. Using a cutoff for SpeB-positivity of 3% azocasein digestion activity, relative to a reference strain (Alab49; impetigo isolate) having high levels of SpeB activity, the two assays were concordant for 150 (98.7%) of the isolates tested. The range of % azocasein digestion activity was rather wide among GAS isolates that produced SpeB and shared the same *emm* type (S3 Table). Taken together, the three assays—Columbia-SM agar, CBrothMg-C agar, C-Broth-azocasein—yield highly concordant findings.

### SpeB production among GAS isolates in accordance with *emm* type

Among the entire study sample of 322 isolates, nine *emm* types are represented by ≥10 isolates. For these highly prevalent *emm* types, SpeB-non-producers ranged from 6 to 100% of the isolates (S1 Fig).

Of the ten *emm18* URT isolates under study, all are SpeB-non-producers. Yet, these 10 isolates are represented by five *emm* subtypes and thereby, consist of a mix of distinct genotypes. A critical question is whether the SpeB-non-producer phenotype that is highly prevalent among *emm18* strains is a consequence of strong selection. The 150 nt region encoding *emm* type-specific determinants was analyzed for genetic change among the *emm18* isolates. Single nucleotide polymorphisms were identified at 17 of the 150 nt sites, which together yield 12 amino acid substitutions (data not shown). Irrespective of whether the underlying mechanism for genetic change is mutation, recombination or a combination of both, a role for strong (diversifying) selection acting on the *emm18* type-specific coding region is evident. Furthermore, the rate of random genetic change observed for *emm* appears to be sufficiently high to readily allow for reversion to a SpeB-producer phenotype—which could arise via mutation in any one of several genes having many more potential target sites (reviewed in [15])—if indeed such a mutation conferred a strong adaptive advantage to the organism. This evolutionary argument supports the notion that the SpeB-non-producer phenotype is directly or indirectly linked to a strong fitness advantage for *emm18* organisms infecting the URT.

Eighteen *emm1* invasive disease isolates were examined in this study and of these, 17 were SpeB-producers on Columbia-SM, but fewer (N = 13) were full-fledged SpeB-producers on CBrothMg-C agar (S1 Table). This relatively high discordance between the two casein digestion assays for *emm1* invasive isolates—Columbia versus C-broth—may be an indication that SpeB activity in *emm1* strains is influenced by unspecified factors in the growth medium. Conceivably, other studies investigating SpeB activity by invasive *emm1* isolates, which used different culture medium, are similarly impacted [16, 21, 28].

**Fig 1:**
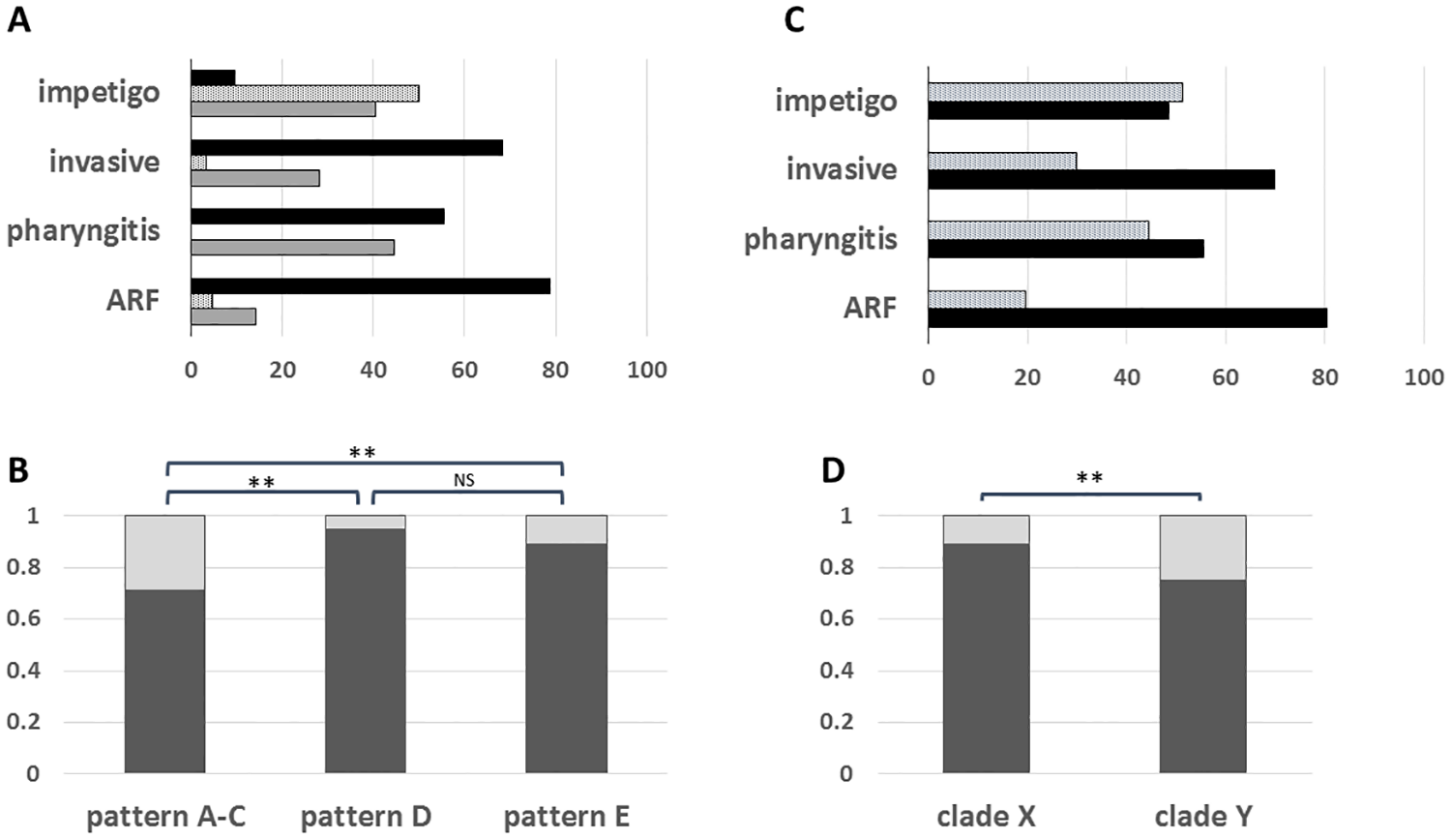
Study sample composition and SpeB production according to *emm* pattern group and *emm* clade. All GAS isolates under study were assigned to an *emm* pattern group and *emm* clade, based on their *emm* type. Panels A and C: The *emm* pattern (panel A: pattern A-C, black; pattern D, speckled; pattern E, gray) and clade (panel C: clade X, speckled; clade Y, black) distributions (%) of each disease-defined subset of GAS strains is shown. Panels B and D: The fractional distributions of SpeB producers (dark gray) and SpeB non-producers (light gray) are plotted. SpeB phenotype is based on the Columbia-SM agar assay. The statistical significance of differences was tested using the Fisher exact test (2-tailed; ** for *p* < 0.01).

Most *emm* types can be assigned to an *emm* pattern group based on the number and arrangement of *emm* and *emm*-like genes, as defined by the 3' end region sequence encoding the cell wall-spanning domain of M and M-like proteins [22, 29–31]. Importantly, *emm* pattern groupings display highly significant correlations with cases of pharyngitis versus impetigo, whereby group *emm* pattern A-C strains are considered to be "throat specialists", pattern D strains are "skin specialists", and pattern E strains are "generalists" [9, 12]. Based on *emm* type, an *emm* pattern group could be predicted for nearly all (99.7%) of the GAS isolates of this study [29] (S1 Table). For this sample set, *emm* pattern A-C isolates accounted for the majority of pharyngitis isolates (55%), but only a small minority of impetigo isolates (9%; Fig 1A); *emm* pattern A-C isolates were even more prevalent among ARF and invasive disease isolates (79 and 68%, respectively). Pattern A-C strains had the highest proportion of SpeB-non-producers (29%), as compared to *emm* pattern D and E strains (5 and 11%, respectively; Fig 1B). The difference in SpeB activity among *emm* pattern A-C isolates versus pattern D or E isolates is highly significant (*p* < 0.001; Fisher's exact test, 2-tailed). Previous findings on a smaller sample set of GAS strains (N = 40) showed similar trends, with mean average SpeB activity levels of *emm* pattern D > pattern E > pattern A-C strains [27].

In addition to *emm* pattern grouping, *emm* types can be assigned to phylogenetically-based *emm* cluster groups, of which there are two major clades [32]. Most pattern A-C *emm* types (throat specialists) fall into clade Y and most pattern E *emm* types (generalists) fall into clade X, whereas pattern D isolates (skin specialists) are split between the two clades [9, 32]. Clade Y is the dominant clade for GAS isolates of this study, accounting for 47, 55, 70 and 80% of impetigo, pharyngitis, invasive disease and ARF isolates, respectively (Fig 1C). The proportion of clade Y pharyngitis isolates for the sample set of this study (55%; N = 146) closely parallels the 52% of pharyngitis isolates collected in North America over a recent seven-year period (N = 7,040) that are clade Y [33, 34]. Considering all GAS isolates, the difference between SpeB-producers and non-producers having *emm* types assigned to clade X versus clade Y is highly significant (*p* = 0.0022; Fisher's exact test, 2-tailed), whereby 11 and 25% of clade X and Y isolates, respectively, lack SpeB activity (Fig 1D).

#### Within-patient (lack of) heterogeneity in SpeB phenotype

During experimental infection in mice following large inoculum doses of GAS, SpeB-non-producers can arise from SpeB-producers via mutations in transcriptional regulatory genes, whereby the switch between SpeB-producer and non-producer corresponds to the transition between localized and invasive GAS infection [13, 19, 20, 35]. Combined with the finding of heterogeneity in SpeB phenotype among GAS isolates sharing the same *emm* type (S1 Fig), it was of interest to determine if a mixture of SpeB phenotypes could be readily detected within individual pharyngitis patients (CMH series of GAS isolates; S1 Table). Data show that all 68 patients having >1 β-hemolytic colony from an oropharyngeal swab yielded homogeneous SpeB phenotypes; 60 patients yielded ≥10 β-hemolytic colonies and 33 patients had ≥25 β-hemolytic colonies (Table 3). In addition, there was no apparent difference between SpeB-producers and non-producers in terms of the number of β-hemolytic colonies recovered from cultured throat swabs (t = 0.68; unpaired Student t-test, 2-tailed). Thus, if SpeB-non-producers arise from infections with SpeB-producing organisms, or vice versa, their numbers are below the limits of detection of this study; those approximate values are calculated as <1 per 1,055 colonies for a SpeB-producer → non-producer transition, and <1 per 341 colonies for a SpeB-non-producer → producer transition (data not shown).

**Table 3:** Phenotype homogeneity among single colony picks from oropharyngeal swabs taken from pediatric patients with pharyngitis.

No. of colony picks per patient	No. of patients yielding 100% SpeB-producer colony picks*	No. of patients yielding 100% SpeB-non-producer colony picks*	No. of patients yielding a mixture of SpeB- producer and non-producer colony picks
2 to 10	7	1	0
11 to 24	21	6	0
≥ 25	24	9	0

* Columbia-SM agar assay

To address the possibility that bacterial cells within a colony are heterogeneous in their SpeB phenotype, 8 colonies arising from oropharyngeal swabs of 8 different pharyngitis patients were passed through 5 μm filters (which excludes long chains), plated onto blood agar, and colony picks tested for SpeB activity on Columbia-SM agar. All colony picks originating from a single throat swab colony were homogeneous in their SpeB phenotype (Table 4).

**Table 4:** Phenotypic homogeneity among organisms within single colonies from oropharyngeal swabs taken from pediatric patients with pharyngitis.

GAS strain	*emm* subtype	No. of CFUs following colony filtration (x 10^3^)	No. of CFUs screened	Predominant SpeB phenotype	% of CFUs expressing the predominant SpeB phenotype
CMH100	6.4	27.4	499	producer	100
CMH103	12.0	60.0	50	producer	100
CMH109	87.0	6.2	940	producer	100
CMH113	3.91	3.9	50	non-producer	100
CMH119	2.0	1.2	50	producer	100
CMH120	5.14	13.9	50	producer	100
CMH125	1.0	13.3	49	producer	100
CMH135	1.0	6.8	50	non-producer	100

CFU, colony forming unit

### SpeB phenotype differences are due to transcription control

**Fig 2:**
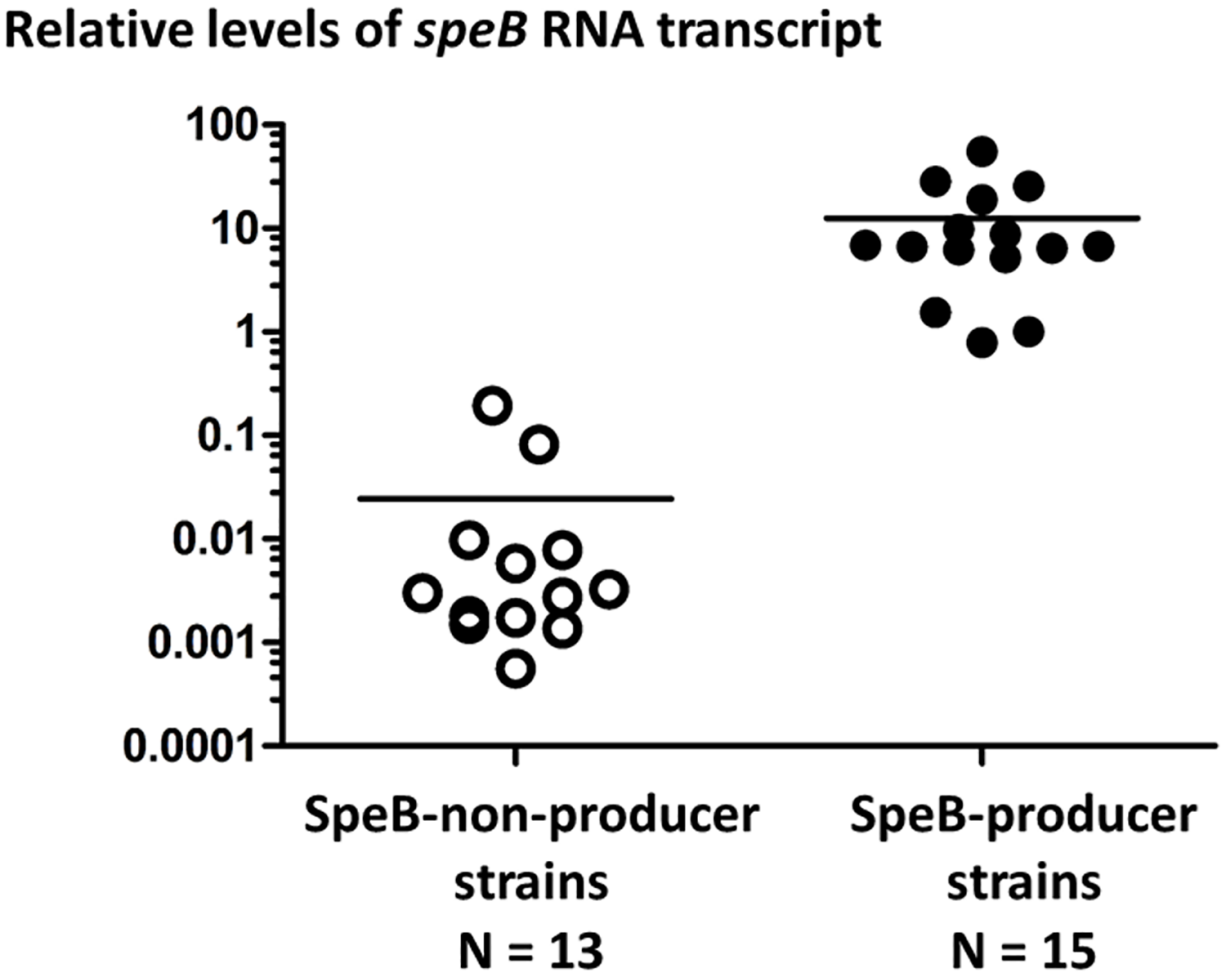
Relative *speB* RNA transcript levels for SpeB-producer and non-producer GAS strains. Normalized (log_10_) relative levels of *speB* transcript for SpeB-producer and non-producer GAS strains are plotted. A single RNA/cDNA sample of the SpeB-producer strain CT02-99 (*emm6*) was chosen as the reference for normalization; its relative *speB* transcript level was adjusted to 1. Mean average values for each group are indicated (bars).

Many studies on naturally-arising mutations affecting SpeB activity (e.g., [13, 36, 37]) in clinical isolates indicate that the molecular basis for differential SpeB casein-digesting activity is typically due to *speB* transcription; *speB* is essentially a core gene of GAS. Using quantitative RT-PCR based on RNA recovered from bacterial cultures grown to stationary phase in C-broth culture medium, normalized values for relative RNA levels of *speB* transcript were compared for 15 strains that displayed caseinolytic activity (on Columbia-SM agar), and 13 strains that were classified as SpeB-non-producers, collectively representing *emm3*, *emm5*, *emm6* and *emm17* isolates of ≥ 17 *emm* subtypes. The difference in relative *speB* transcript levels for SpeB-producers versus non-producers is highly significant (Fig 2; t = 0.0047, unpaired Student t-test, 2-tailed, with Welch’s correction; *p* < 0.0001, Mann-Whitney U-test, 2-tailed). The normalized average mean value for *speB* RNA transcript levels from SpeB-producer strains exceeds that of non-producer strains by >500-fold. In summary, for the 28 GAS strains examined, SpeB-mediated caseinolytic activity observed after 48 hours of culture on a solid surface (agar) is highly correlated with relative RNA transcript levels for *speB* following 16 hours of culture in liquid broth medium.

## Discussion

As a species, GAS are extraordinary in the complexity and diversity of their genotypes, phenotypic expression, and the wide range of human diseases they can cause. Untangling these relationships, via identifying epidemiologic correlates between microbial genotypes, phenotypes and clinical disease, can often lead to a greater understanding of the pivotal molecular interactions between agent and host. The findings of this report on the strong correlations between the SpeB activity phenotype and different human diseases caused by GAS, although imperfect, may provide such a foundation. For ARF, the defining attributes of a “rheumatogenic” strain have remained elusive, and the finding for a relative lack of SpeB activity among ARF-associated isolates may provide a molecular handle.

The highly significant association between SpeB-non-producers and ARF provides support for a novel hypothesis: That the ability of GAS to trigger ARF is due, in part, to a phenotype that is related to depressed SpeB activity. However, many ARF-associated isolates produce SpeB and therefore, a lack of SpeB can, at best, provide only a partial explanation for rheumatogenic potential. It is noteworthy that the *emm18* (100% SpeB-non-producers) and *emm3* (50% SpeB-non-producers) organisms recovered from the URT in this study (i.e., pharyngitis and ARF-associated combined; S1 Table) represent *emm* types that were highly prevalent in ARF outbreaks within the United States during the 1980s [38, 39].

A relative lack of SpeB activity may have direct and indirect effects on the pathogenesis of ARF. An indirect effect might arise from the orchestrated expression of other genes having a role in ARF (e.g., CovRS, RopB) (reviewed in [13, 15]). A direct effect for a lack of SpeB might entail the sparing of extracellular GAS proteolytic targets (reviewed in [13, 14]). M protein elicits an autoimmune response to cardiac myosin through antigenic mimicry [40], but it is also cleaved by SpeB; conceivably, unmodified M protein may yield a more potent cross-reactive immune response. SpeB degrades superantigens produced by GAS [17], and it has been postulated that superantigens play a role in autoimmune processes.

SpeB also cleaves streptolysin O (SLO) and deoxyribonucleases (DNases) produced by GAS. The revised Jones criteria [3] used for establishing a diagnosis of ARF requires evidence for a recent GAS infection, which is usually satisfied by elevated anti-SLO (ASO) or anti-DNaseB (ADB) titers when GAS is not recovered from oropharyngeal swabs, as is very often the case. A plausible consequence of infections with SpeB-non-producers is that ASO and ADB titers are elevated because SLO and DNaseB remain fully intact. This argument raises the possibility that cases of ARF triggered by SpeB-producers are under-diagnosed. Since the URT infection preceding ARF is often clinically inapparent, the possibility should also be considered that a lack of SpeB production results in fewer overt symptoms of pharyngitis. If correct then this, in turn, would provide an adaptive advantage to SpeB-non-producers because they would be more likely to escape diagnosis and therefore antibiotic treatment.

Well-documented ARF-associated GAS isolates are not readily available and difficult to obtain, largely because the inciting GAS infection is usually resolved by a host immune response well before the first acute clinical signs of autoimmune disease appear. The ARF-associated organisms selected for this analysis (S1 Table) were derived from institutional or community outbreaks that were well-studied, or individual patients that were carefully tracked; like the pharyngitis and invasive disease isolates, all were recovered from the United States. However, ARF is highly prevalent in many resource-poor regions of the world [1], where the circulating GAS strains differ markedly in *emm* type from GAS of resource-rich regions, and impetigo is often endemic [41, 42]. Whether ARF-associated organisms from resource-poor regions exhibit significantly lower levels of SpeB activity, when compared to the larger pool of circulating strains, remains to be established.

The findings of this report on SpeB activity and disease association confirm some previous studies. That impetigo isolates have significantly higher levels of SpeB activity than pharyngitis isolates had been noted in a small-scale study, wherein the role of SpeB in impetigo was experimentally validated as well [43]. In the present study, ~20% of both pharyngitis and invasive disease isolates, each represented by ≥ 20 *emm* types, lacked SpeB activity, a finding that closely parallels the data on several thousand pharyngitis and/or invasive disease GAS isolates represented by four *emm* types [21]. In a prior study, *emm1* isolates that were stratified according to severe versus non-severe invasive disease showed statistically significant differences in SpeB production, whereby ~40% of severe invasive disease isolates were SpeB-deficient [16]. The data of this study were unable to replicate those latter findings, however, the sample size for *emm1* invasive disease isolates may have been too small to distinguish among clinical subgroups. A role for a lack of SpeB in GAS dissemination to systemic tissue sites has also been experimentally validated [13, 18–20].

Population studies by other investigators studying SpeB activity focused on invasive and/or URT isolates sharing the same *emm* type [16, 21]. A distinct advantage of the genetically diverse collection of isolates used in this study is that biological tendencies which are broadly associated with a disease group may be more readily captured. For many *emm* types, organisms tend to cause only a subset of the numerous GAS disease types; for e.g., the highly rheumatogenic *emm18* organisms are (relatively) rarely recovered from cases of pharyngitis or invasive disease [34, 44–47].

Among the SpeB-producer strains, the biological implications of “lower” versus “higher” levels of SpeB activity, if any, are not known. It is plausible that relatively low levels of SpeB activity leads to degradation of GAS extracellular proteins while leaving more distal host tissue proteins largely intact. Although the subset of 152 strains tested for azocasein digestion do not fully reflect the larger set of 322 isolates in terms of genetic diversity (Table 1), some insights may be gleaned from the data. Comparisons of % azocasein digestion activity for SpeB-producer strains reveal no significant difference for ARF versus pharyngitis isolates, but do show statistically significant differences for impetigo versus either ARF or pharyngitis isolates (*t* < 0.05; unpaired student t-test with Welch’s correction, 2-tailed). Thus, the absolute level of SpeB activity may further distinguish URT from superficial skin infections, consistent with previous findings [27]. Future studies can more carefully explore biologically significant thresholds for SpeB activity and/or *speB* transcript levels.

Each GAS strain appears to have a strong tendency towards causing only a limited subset of the human diseases that have been associated with this species. The genotypic diversity among GAS isolates is partly manifest by a very large number of accessory genes, several of which also display extensive nt sequence divergence [12, 48, 49]. Equally complex is the vast array of intersecting transcriptional networks that coordinate the expression of virulence factor genes. Because *speB* expression is regulated via numerous pathways, SpeB activity provides a simple output for measuring the transcriptional state(s) of the cell. Strong tendencies for SpeB activity versus SpeB deficits among collections of clinically-defined isolates, as revealed in this report, may in turn reflect distinguishing molecular pathways that contribute to disease phenotypes and pathogenesis.

## Materials and methods

### Strain sampling

GAS isolates were chosen using a strategy that aimed to include strains representative of the human host clinical sub-populations, coupled with maximizing genetic diversity. For pharyngitis isolates from a population-based collection (N = 78; Bristol, CT, 2001–2002) [44], one isolate of each *emm* subtype [49] was selected; if there were multiple isolates of a given *emm* subtype, ~33% of isolates (range, 25–40%) of that *emm* subtype were chosen; overall ≥30% of the isolates of a given *emm* type were sampled. For pharyngitis isolates whose *emm* types were unknown at the onset of the study (N = 68 patients; Chicago, 2012), between 2 and 53 colony picks of β-hemolytic colonies derived from pediatric throat swabs plated on blood agar were collected for analysis; group A carbohydrate was confirmed by latex bead agglutination, and the *emm* type and subtype was ascertained by sequence-based typing of representative colony picks; one representative isolate from each patient was used for further study, unless otherwise noted; the phenotypic homogeneity of multiple colony picks from the same throat swab is demonstrated in Table 3. All ARF-associated isolates (N = 42; USA, 1933–1989) were recovered from the upper respiratory tract (URT; most were previously characterized [50–52]); sources for ARF-associated isolates are The Rockefeller University Hospital (RS or RP, rheumatic patient series; N = 15, NY), the Great Lakes Naval Training Station (N = 8; IL), others from the Lancefield collection (N = 4; NY), WHO-Minneapolis (N = 12; USA) and the C.D.C. (N = 3); ≤ 2 ARF-associated isolates from the same collection (i.e., strain source and sample type) and sharing an *emm* type were chosen. For impetigo isolates of a population-based collection (N = 58; tropical Australia, 1994–1996) [26, 53], ≥ 1 isolate of each *emm* subtype was sampled; if there were 2 to 6 isolates of a given *emm* subtype, 2 isolates were sampled, and if there were 7 to 10 isolates of a given *emm* subtype, 3 isolates were sampled. An additional 16 impetigo isolates, each having a distinct *emm* type, are also included (from USA, Trinidad and Czech Republic; 1971–1988) [50]. Invasive disease isolates were collected from normally sterile tissue sites of patients in CT hospitals over a 6-month period in 1995 as previously described [23]; included in this study are 60 of the 64 original GAS isolates that were reported (4 cultures had been lost); all available isolates were included in the analysis.

### *emm* type determination

*emm* subtype (which is roughly equivalent to partial allele) was assigned according to [49].

### Agar-based casein digestion assays

Modified Columbia agar with skim milk (Columbia-SM agar) was prepared as follows: 0.5X Columbia agar base (Difco-BBL), 3% w/v skim milk powder (Difco-BBL) and additional Bacto-agar for 1.5% w/v final concentration. Following GAS inoculation of agar (by short stab of a colony pick derived from a blood agar plate, or surface plating of 2 μl of 10-fold concentrated C-broth culture that had been grown overnight) and 48 h incubation at 37°C, SpeB-producers had average zones of clearance ≥6.0 mm, whereas SpeB-non-producers had average zones of clearance of 0 to 1.0 mm. All GAS isolates were tested in multiple replicates.

For CBrothMg-C agar, C-broth was prepared by the standard method [27], except with 7.5 mM MgSO_4_, 3% w/v casein (Acros Organics) and 1.5% w/v Bacto-agar added. Following GAS inoculation of agar plates (done by plating 2 μl of a 10-fold concentrated C-broth culture grown overnight) and 48 h incubation at 37°C, SpeB-producers had average zones of clearance ≥4.0 mm, whereas SpeB-non-producers had average zones of clearance of 0 to 1.0 mm. Only a subset of pharyngitis and impetigo isolates were tested using CBrothMg-C agar (S1 Table), and the % of SpeB-non-producers was extrapolated to the whole sample set. Intermediate zones of clearance were scored as "weak.”

### Azocasein digestion assay

The azocasein digestion assay was performed using culture supernatants, as previously described [27], following growth of GAS in C-broth for 16 h at 37°C; data is expressed as % SpeB activity relative to the control strain Alab49. Organisms were tested in multiple replicates, and % SpeB activity values averaged.

### Quantitative PCR (qPCR)

RNA was purified from cells grown 16 h at 37°C in C-broth, and the cDNA generated was used as a template for quantitative PCR, according to [54]. Both the *recA* and *gyr* housekeeping genes were used to calculate relative RNA transcript levels for the *speB* target gene, and values were averaged. One SpeB-producer strain was chosen as the reference for normalization of relative *speB* RNA transcript levels for all other strains of the dataset. Each gene was tested in triplicate; independent RNA preparations (up to four) were generated for many strains, and relative *speB* transcript levels (normalized values) were reported as the mean average. Oligonucleotide primers are as follows: *recA*-forward, 5’-ATTGATTGATTCTGGTGCGG; *recA*-reverse, 5’-ATTTACGCATGGCCTGACTC; *gyr*-forward, 5’-CGATGCCAGTCAAATTCAGG; *gyr*-reverse, 5’-CCCAGACTAAATGATGCAAACCC; *speB*-forward, 5’-TGTCGGTAAAGTAGGCGGAC; *speB*-reverse, 5’-GAGCTGAAGGGTTTAGTGCG.

## Supporting information

S1 Table Epidemiological and experimental findings for 322 GAS isolates. (XLSX)

S2 Table Distribution of shared *emm* types among clinical disease groups. (PDF)

S3 Table Comparison of SpeB phenotype assignment to range of activity by the azocasein broth assay. (PDF)

S1 Fig Heterogeneity in SpeB phenotype among all clinical isolates sharing the most common *emm* types. The fractional distribution of SpeB-producers (dark gray) and SpeB-non-producers (light gray) is plotted in accordance with *emm* type, for all isolates of the most common *emm* types: *emm1* (N = 46 isolates); *emm2* (N = 11); *emm3* (N = 29); *emm4* (N = 11); *emm6* (N = 20); *emm12* (N = 27); *emm18* (N = 11); *emm28* (N = 17); *emm89* (N = 10). SpeB phenotype is based on the Columbia-SM agar assay.
(PDF)
