# Supplementary material for: Differences in SpeB protease activity among group A streptococci associated with superficial, invasive, and autoimmune disease
Source: PLoS One. 2017 May 17;12(5):e0177784. doi: 10.1371/journal.pone.0177784 (PMC5435240; doi:10.1371/journal.pone.0177784)
Supplement: S2 Table — (PDF) [file pone.0177784.s002.pdf]

S2 Table. Distribution of shared *emm* types among clinical disease groups.

Disease	No. of isolates	No. of *emm* types represented	% of disease isolates having an *emm* type overlapping with *emm* types of isolates from cases of:			
			Pharyngitis	ARF	Invasive	Impetigo
Pharyngitis	146	21	100	66	84	15
ARF	42	20	57	100	57	7
Invasive	60	20	88	9	100	5
Impetigo	74	39	19	9	9	100
