# Supplementary material for: Differences in SpeB protease activity among group A streptococci associated with superficial, invasive, and autoimmune disease
Source: PLoS One. 2017 May 17;12(5):e0177784. doi: 10.1371/journal.pone.0177784 (PMC5435240; doi:10.1371/journal.pone.0177784)
Supplement: S3 Table — (PDF) [file pone.0177784.s003.pdf]

S3 Table. Comparison of SpeB phenotype assignment to range of activity by the azocasein broth assay.

emm type	% azocasein activity for SpeB-non-producer strains [a]	% azocasein activity for SpeB-producer strains [a]
2	0	38 - 86
3	0	29 - 95
4	0	88 - 117
5	n.d.	40 - 79
6	0 - 0.5	5 - 92
18	0	n.d.

[a] Based on findings from Columbia-SM agar assay
