# Supplementary material for: Differences in SpeB protease activity among group A streptococci associated with superficial, invasive, and autoimmune disease
Source: PLoS One. 2017 May 17;12(5):e0177784. doi: 10.1371/journal.pone.0177784 (PMC5435240; doi:10.1371/journal.pone.0177784)
Supplement: S1 Fig — The fractional distribution of SpeB-producers (dark gray) and SpeB-non-producers (light gray) is plotted in accordance with emm type, for all isolates of the most common emm types: emm1 (N = 46 isolates); emm2 (N = 11); emm3 (N = 29); emm4 (N = 11); emm6 (N = 20); emm12 (N = 27); emm18 (N = 11); emm28 (N = 17); emm89 (N = 10). SpeB phenotype is based on the Columbia-SM agar assay. (PDF) [file pone.0177784.s004.pdf]

**S1 Figure. Heterogeneity in SpeB phenotype among all clinical isolates sharing the most common *emm* types.** The fractional distribution of SpeB-producers (dark gray) and SpeB non-producers (light gray) is plotted in accordance with *emm* type, for all isolates of the most common *emm* types: *emm1* (N = 46 isolates); *emm2* (N = 11); *emm3* (N = 29); *emm4* (N = 11); *emm6* (N = 20); *emm12* (N = 27); *emm18* (N = 11); *emm28* (N = 17); *emm89* (N = 10). SpeB phenotype is based on the Columbia-SM agar assay.

Figure S1

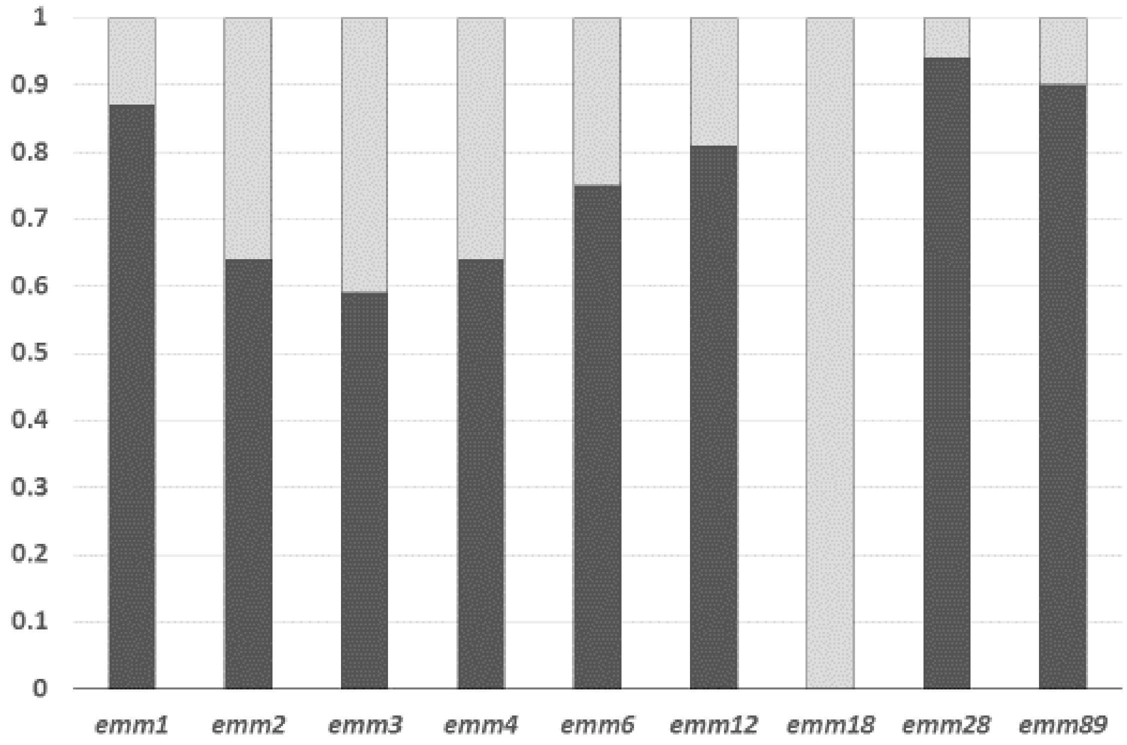

1
0.9
0.8
0.7
0.6
0.5
0.4
0.3
0.2
0.1
0
emm1
emm2
emm3
emm4
emm6
emm12
emm18
emm28
emm89
